# A Bioinformatics-Based Analysis of an Anoikis-Related Gene Signature Predicts the Prognosis of Patients with Low-Grade Gliomas

**DOI:** 10.3390/brainsci12101349

**Published:** 2022-10-05

**Authors:** Songyun Zhao, Hao Chi, Wei Ji, Qisheng He, Guichuan Lai, Gaoge Peng, Xiaoyu Zhao, Chao Cheng

**Affiliations:** 1 Department of Neurosurgery, Wuxi People’s Hospital Affiliated to Nanjing Medical University, Wuxi 214000, China; 2 Clinical Medicine College, Southwest Medical University, Luzhou 646000, China; 3 Department of Epidemiology and Health Statistics, School of Public Health, Chongqing Medical University, Yixue Road, Chongqing 400016, China; 4 Department of Neurosurgery, The First Affiliated Hospital of Xinjiang Medical University, Urumqi 830054, China

**Keywords:** anoikis, low-grade glioma, signature, prognosis, immune microenvironment

## Abstract

Low-grade glioma (LGG) is a highly aggressive disease in the skull. On the other hand, anoikis, a specific form of cell death induced by the loss of cell contact with the extracellular matrix, plays a key role in cancer metastasis. In this study, anoikis-related genes (ANRGs) were used to identify LGG subtypes and to construct a prognostic model for LGG patients. In addition, we explored the immune microenvironment and enrichment pathways between different subtypes. We constructed an anoikis-related gene signature using the TCGA (The Cancer Genome Atlas) cohort and investigated the differences between different risk groups in clinical features, mutational landscape, immune cell infiltration (ICI), etc. Kaplan–Meier analysis showed that the characteristics of ANRGs in the high-risk group were associated with poor prognosis in LGG patients. The risk score was identified as an independent prognostic factor. The high-risk group had higher ICI, tumor mutation load (TMB), immune checkpoint gene expression, and therapeutic response to immune checkpoint blockers (ICB). Functional analysis showed that these high-risk and low-risk groups had different immune statuses and drug sensitivity. Risk scores were used together with LGG clinicopathological features to construct a nomogram, and Decision Curve Analysis (DCA) showed that the model could enable patients to benefit from clinical treatment strategies.

## 1. Introduction

Glioma is a relatively common primary tumor in the brain that originates mainly from glial cells in the brain tissue, and about one-third of gliomas are low-grade gliomas (LGGs) [1]. low-grade glioma is a diffusely infiltrating, slow-growing glial brain tumor that tends to have extensive genetic and transcriptional heterogeneity [2]. According to the Cancer Genome Atlas Project classification, “low-grade glioma” has taken the place of the phrase “lower grade glioma,” which was formerly often used to refer to grade 2 gliomas [3,4]. LGG is usually considered to have a benign course; however, diffuse low-grade gliomas (LGG) naturally transform into malignant high-grade gliomas, and once recurring and progressing to high-grade gliomas, they can greatly limit a patient’s survival [5]. The poor prognosis and mortality associated with gliomas are mainly due to the highly aggressive and mobile nature of the tumor cells, which can spread widely into the surrounding brain tissue [6]. To date, the molecular mechanisms of glioma invasion and migration are not fully understood [7]. The epithelial-mesenchymal transition (EMT) of glioma cells is a significant component contributing to the aggressiveness of high-grade gliomas. For prompting clinical interventions to halt the growth of the disease, more novel biomarkers are urgently required at an early stage to predict the prognosis of glioma patients.

In the absence of extracellular matrix (ECM) attachment or when adhered to inappropriate sites, cells undergo a specific type of apoptosis called anoikis [8,9]. Anoikis acts as an important defense for the organism by preventing shedding cells from re-adhering to incorrect locations and preventing their growth [8,10]. Tumor cells have been found to resist anoikis in a variety of ways, such as secreting growth factors, activating pro-survival signaling pathways, or altering the expression pattern of integrins by cells [11]. Resistance to anoikis is becoming a hallmark of cancer cells and contributes to the formation of distant organ metastases [12,13]. However, few studies focused on the relationship between the process of anoikis and distant metastasis of LGGs.

Therefore, we developed a prognostic scoring model based on anoikis-related genes (ANRGs), and under this risk score, we further investigated the relationship between ANRGs and the immune microenvironment, immune checkpoint blockers (ICB) response, and chemotherapy sensitivity. We aim to demonstrate the value of ANRGs for assessing the prognosis of LGG patients through a comprehensive analysis of genomic data and to develop a novel signature based on ANRGs that can accurately predict the prognosis of LGG patients and characterize the immune landscape, thereby improving treatment options.

## 2. Materials and Methods

### 2.1. Gene Expression and Clinical Data Acquisition

**Figure 1:**
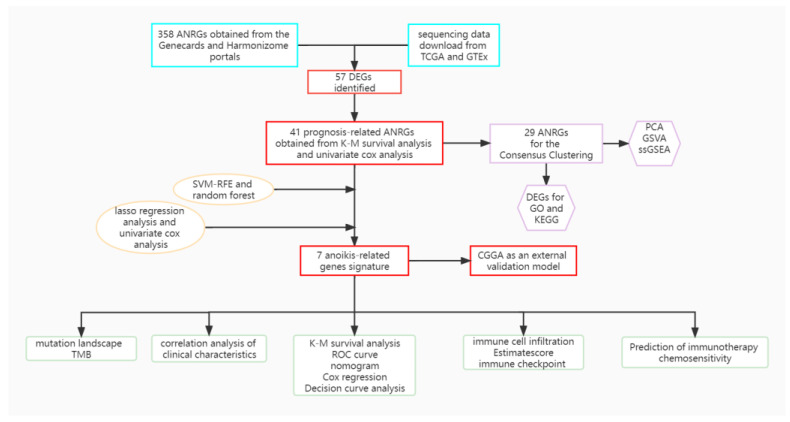
A detailed flow chart about the study of ANRGs in LGG.

Gene expression profiles of TCGA-LGG were downloaded from the UCSC Xena website (https://xena.ucsc.edu/, accessed on 20 August 2022). 529 LGG tissue samples were available in the TCGA(The Cancer Genome Atlas) cohort. Gene expression profile data for the 443 patients with low-grade gliomas in the validation model was obtained from the China Glioma Genome Atlas (CGGA) data portal (http://www.cgga.org.cn/, accessed on 20 August 2022). In addition, normal control samples were obtained from The Genotype-Tissue Expression (GTEx) website (https://www.gtexportal.org/, accessed on 20 August 2022). FPKM data was finally transformed into transcript per million (TPM). Batch corrections and integrations of the two sets of gene expression data were performed with the “limma” and “sva” [14] packages. A detailed flow chart is shown in Figure 1.

### 2.2. Acquisition of ANRGs

A total of 358 ANRGs were downloaded from the GeneCard database [15] (https://www.genecards.org/, accessed on 15 August 2022) and Harmonizome portals (https://maayanlab.cloud/Harmonizome/, accessed on 15 August 2022) [16]. Differential expression analysis of differentially expressed genes (DEGs) was performed for the TCGA cohort and different subgroups using the R software “limma” package with | log2FC | > 1.0 and FDR < 0.05 as thresholds.

### 2.3. Functional Enrichment Analysis

We downloaded “c2. cp.kegg.v7.4. symbols. gmt” from the MSigDB database to carry out GSVA analysis. The “GSVA” R package was used to perform GSVA enrichment analysis [17]. In addition to this, functional enrichment analysis was performed by the “clusterProfiler” package in the R software (Version 4.1.0) (https://www.r-project.org, accessed on 1 September 2022), including the Kyoto Encyclopedia of Genes and Genomes (KEGG) and Gene Ontology (GO) analysis.

### 2.4. Consensus Clustering

Consensus clustering was applied to identify distinct anoikis-related patterns relating to the expression of anoikis regulators by the k-means method. Thereafter, Uniform Manifold Approximation and Projection (UMAP) was used to validate the reliability of clustering with the R package “ggplot2”.

### 2.5. Selection of Characteristic Genes

Two machine learning algorithms, random forest and SVM-RFE [18] were used to screen for signature genes. Recursive feature elimination (RFE) in the random forest algorithm is a supervised machine learning method for ranking genes in LGGs. Predictive performance was estimated by tenfold cross-validation, and genes with relative importance > 0.25 were identified as feature genes. SVM-RFE is a small-sample learning method that essentially bypasses the traditional process of induction to deduction and enables efficient “transductive inference” from training to prediction samples, simplifying the usual classification and regression problems.

### 2.6. Development and Validation of Prognostic Signatures

First, accurate models were developed using the R package “sva” to eliminate batch effects between TCGA and CGGA data. Variables with *p*-values < 0.01 were selected for the least absolute shrinkage and selection operator (LASSO) regression analysis, and the number of genes in the final risk model was reduced by the R software package “glmnet”. Then, the genes from the LASSO regression were selected for the multivariate Cox regression analysis and thus constructed according to the following equation: risk score = ∑(ð × Exp), where ð is the corresponding regression coefficient and Exp represents the expression value of each mRNA. All patients were divided into high-risk and low-risk groups according to the median risk score. Survival curves and risk maps were generated to show the survival differences and status of each patient by the R software, “survminer” and “ggrisk” packages. In addition, the CGGA cohort was used as an independent external cohort to validate the utility of the prognostic model.

### 2.7. Estimation of the Tumor Immune Microenvironment of the Prognostic Signature

CIBERSORT and ssGSEA R scripts were used to quantify the relative proportion of infiltrating immune cells [19]. We used CIBERSORT to estimate the proportion of immune cell types between the low-risk and high-risk groups. The sum of all estimated immune cell types scored in each sample equals 1. Meanwhile, spearman rank correlation analysis was applied to explore relationships between risk score values and immune infiltrating cells.

### 2.8. Tumor Immune Cycle and ICB Response

We obtained the cancer-immune cycle-related gene set [20] from the website developed by Xu et al. (http://biocc.hrbmu.edu.cn/TIP/, accessed on 20 August 2022) and a set of genes positively associated with clinical response to the anti-PD-L1 drug (atezolizumab) from Mariathasan’s study features [21].

### 2.9. Construction and Evaluation of a Predictive Nomogram

The nomogram was created using risk ratings and clinicopathological features. For internal validation to confirm the accuracy, the calibration plot was executed. DCA, or decision curve analysis, was used to evaluate the clinical net benefit [22]. In addition, we evaluated the accuracy of risk ratings in predicting 1-year, 3-year, and 5-year OS in LGG patients by plotting subject operating characteristic curves using the R software’s (Version 4.1.0) (https://www.r-project.org, accessed on 1 September 2022) “timeROC” package.

### 2.10. Tumor Immune Single Cell Hub Database

An extensive single-cell RNA-seq database devoted to the TME is available online under the name Tumor Immune Single-Cell Hub (TISCH; http://tisch.comp-genomics.org, accessed on 20 August 2022) [23]. Utilizing this database, comprehensive research on TME heterogeneity in diverse data sets and cell types was done.

### 2.11. Statistical Analysis

All analyses were performed using R version 4.1.1, 64-bit6, and its support package. To calculate prognostic values and to compare patient survival in different subgroups in each data set, Kaplan–Meier survival analysis, and the log-rank test was used. The non-parametric Wilcoxon rank sum test was used to test the relationship between the two groups for continuous variables. Kruskal–Wallis test was used as a comparison among more than two groups. Clinical characteristics of the high and low-risk groups were screened for prognostic variables using univariate and multivariate Cox regression (R package ‘survival’). Correlation coefficients were examined using spearman correlation analysis. In all statistical investigations, *p* < 0.05 was considered statistically significant.

## 3. Results

### 3.1. Acquisition of ANRGs

**Figure 2:**
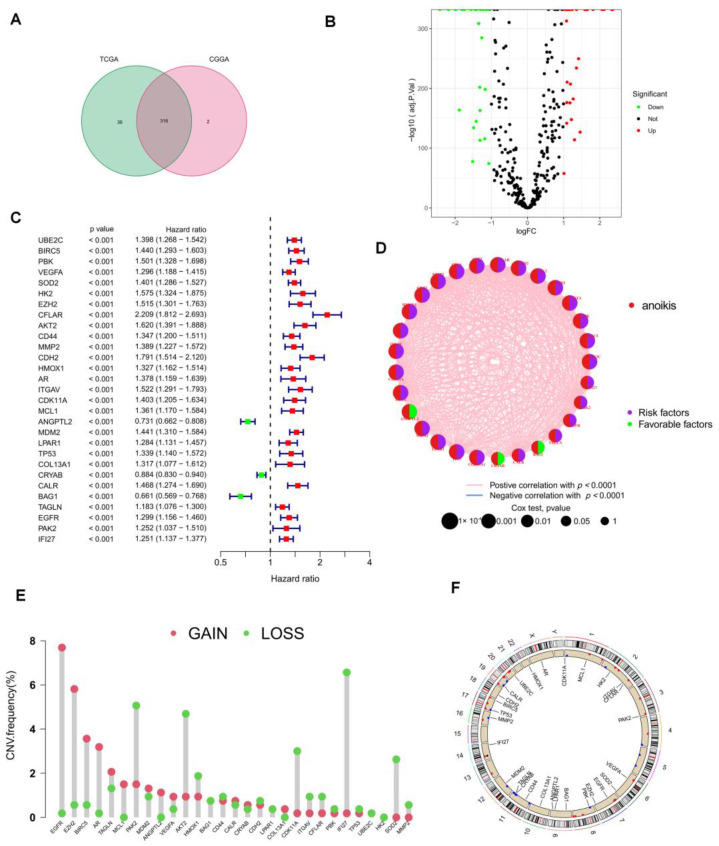
Characteristics of ANRGs in LGG. (**A**) Venn diagram showing the 316 ANRGs found from the TCGA and CGGA cohorts. (**B**) Volcano plot of 57 DEGs in TCGA and GTEx cohort, red for up-regulated, blue for down-regulated genes. (**C**) The forest plot shows the 29 ANRGs (*p* < 0.001) via the univariate Cox regression analysis. (**D**) The network diagram showed the correlations between the top 29ANRGs. The red connecting lines represent positive correlations, while the blue represents negative correlations. (**E**) Copy number variations (CNVs) of 29 ANRGs in TCGA-LGG. (**F**) Localization of 29 ANRGs in chromosomal regions.

The Genecards and Har-monizome portals yielded a total of 358 anoikis-associated genes (Supplementary Table S1), and the TCGA and CGGA cohorts included a combined total of 316 ANRGs (Figure 2A). When compared to normal adjacent tissues, we found 57 differentially expressed genes (DEGs) in the TCGA-LGG and GTEx cohorts, the volcano map of these DEGs is displayed in Figure 2B. To create the new “TCGA-CGGA” cohort, we combined the TCGA-LGG cohort with the CGGA cohort and eliminated the batch effect. 41 of 57 ANRGs were linked with survival and statistically distinct, according to univariate Cox regression analysis (*p* < 0.05, km < 0.05, Supplementary Table S2). The top 29 ANRGs most strongly correlated with prognosis in LGG patients were displayed in the forest plot (*p* < 0.001, Figure 2C). Except for ANGPTL2, CRYAB, and BAG1, 26 genes were associated with poor prognosis. Meanwhile, network plots showed the relationship between the expression levels of the top 29 ranked genes more clearly (Figure 2D). Since LGG patients frequently lost or gained chromosomal regions [24], we downloaded CNV data from the TCGA database to further explore the alteration of these lost apoptosis-related genes on chromosomes and the location of each gene on chromosomes (Figure 2E,F). Figure 2F demonstrates that IFI27 was mostly displayed as a “loss” and was positioned on chromosome 14, while the most substantial changed “gain” of EGFR was located on chromosome 7.

### 3.2. Consistent Clustering of 29 ANRGs in LGG

**Figure 3:**
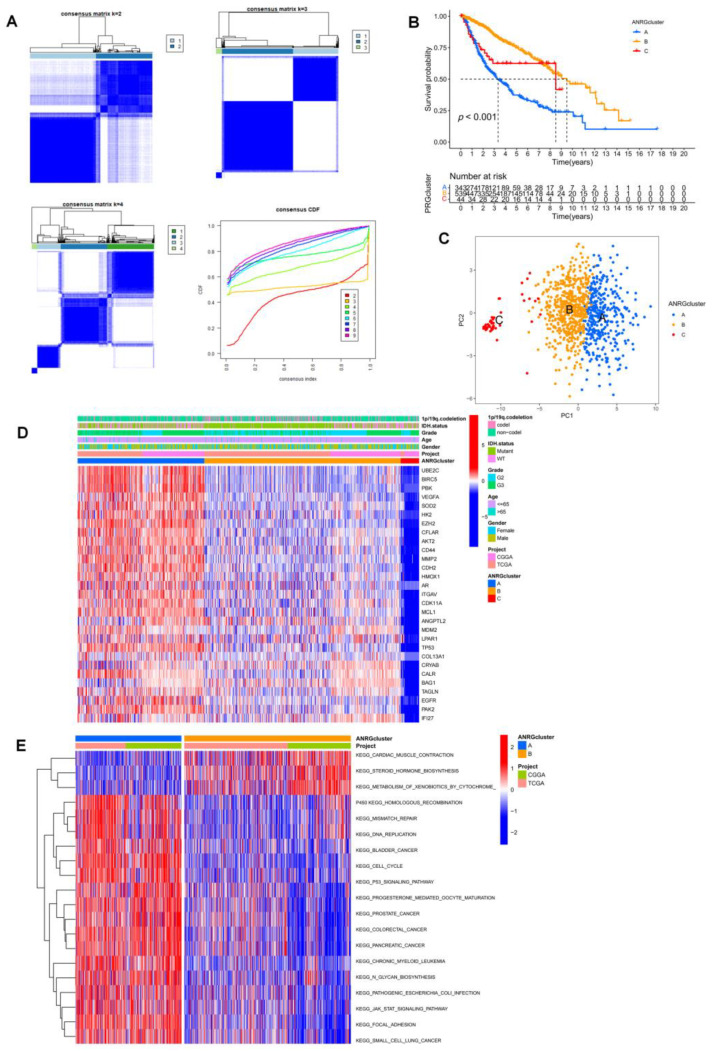
Subgroups of LGGs associated with 29 ANRGs. (**A**) The consensus matrix was obtained by applying consensus clustering when k = 2, 3 and 4. When k = 2, the slope of the CDF curve is the lowest. (**B**) Overall survival of three subtypes (*p* < 0.001). (**C**) PCA distinguished three subtypes based on the expression of ANRGs. (**D**) Heat map of the expression of 29 ANRGs and corresponding clinicopathological features of two subtypes. (**E**) GSVA analysis focused on the differential enrichment of KEGG pathways between clusters A and B.

To comprehend the function of ANRGs in LGG better, we used the ‘Consensus Cluster Plus’ R program to perform consensus clustering based on 29 prognosis-related ANRGs (*p* < 0.001) and the findings of the univariate cox analysis. When k = 3, as in Figure 3A, the cohort could be effectively divided into three subtypes. A substantial difference in prognosis among the three subtypes was revealed by the overall survival analysis (*p* < 0.001, Figure 3B). Its accuracy was examined using principal component analysis (PCA), which was used to classify the data. The findings demonstrated that, at k = 3, the three clusters’ subtypes could be precisely defined (Figure 3C). Heatmaps of ANGs expression and corresponding clinicopathological features of the 3 subtypes indicated that higher expression of ANGRs in cluster A might be associated with a worse prognosis in LGG patients, and interestingly try that very low expression of ANGs in cluster C was not associated with a better prognosis (Figure 3D), so that ANRGs may regulate LGG progression through more complex pathways. We used the GSVA software to concentrate on the differential enrichment of the KEGG pathway between cluster A and cluster B given the obvious disparities among clusters A, B, and C in addition to examining the overall distribution of the 29 ANRGs in clusters (Figure 3E, Supplementary Figure S1). Cluster A with the poorest prognosis was mainly associated with the adhesive junction pathway and some common tumor-associated pathways. In glioma, adhesive linkage-associated proteins can be bound to β-catenin and regulate gene transcription, which ultimately affects the cell cycle, apoptosis, and changes in cytoskeletal structure, affecting cell migration [25].

### 3.3. Immune Infiltration and Differential Gene Expression in the Two Subtype Clusters

**Figure 4:**
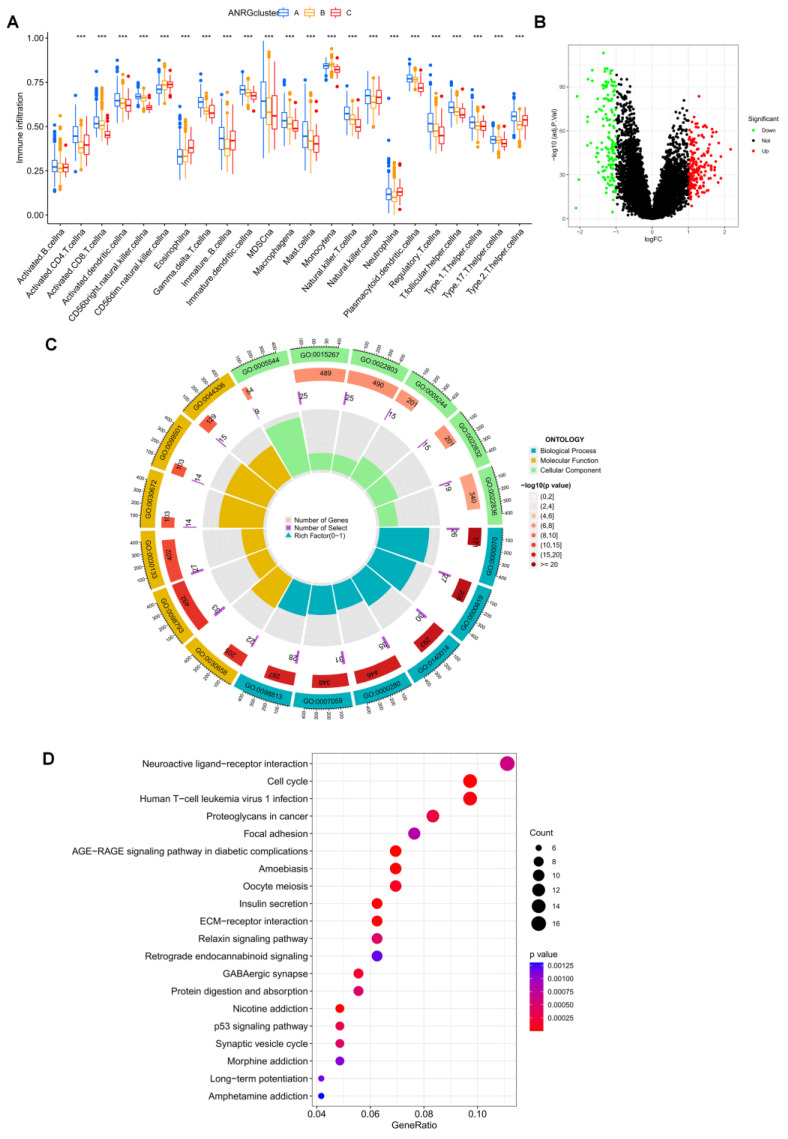
Differences in immune infiltration patterns and enrichment analysis of DEGs in the three subtype groups. (**A**) Immune infiltration patterns of three subtype groups were obtained using ssGSEA. (**B**) Volcano plot of up-and down-regulated DEGs in clusters A and B. (**C**) GO analysis circle diagram of DEGs. (**D**) Bubble diagram of KEGG enrichment analysis of DEGs. *** *p* < 0.001.

A boxplot was utilized to demonstrate the considerable variation in immune cell infiltration levels among the three groupings (Figure 4A). We were surprised to find that almost all percentages of immune cell infiltration were higher in group A than in groups B and C. We performed differential analysis for groups A and B, where patients had the worst survival performance, and volcano plots of the differential analysis were shown in Figure 4B. GO and KEGG enrichment analyses were performed for these differential genes, and these DEGs were associated with a variety of items, including “regulation of trans-synaptic signaling” in the biological process (BP) class, “presynapse” in the cellular component (CC) class, Molecular KEGG results show that these genes are associated with “cell cycle” and “proteoglycans in cancer” (Figure 4C,D), with related evidence that proteoglycans could act as co-receptors for growth factors and co-receptors for cellular matrix proteins, increasing the affinity of adhesion molecules for their specific receptors, and thus proteoglycans play an important role in the acquisition of apoptosis resistance in tumors with anoikis [26,27].

### 3.4. The Development and Validation of an Anoikis-Related Prognostic Signature

**Figure 5:**
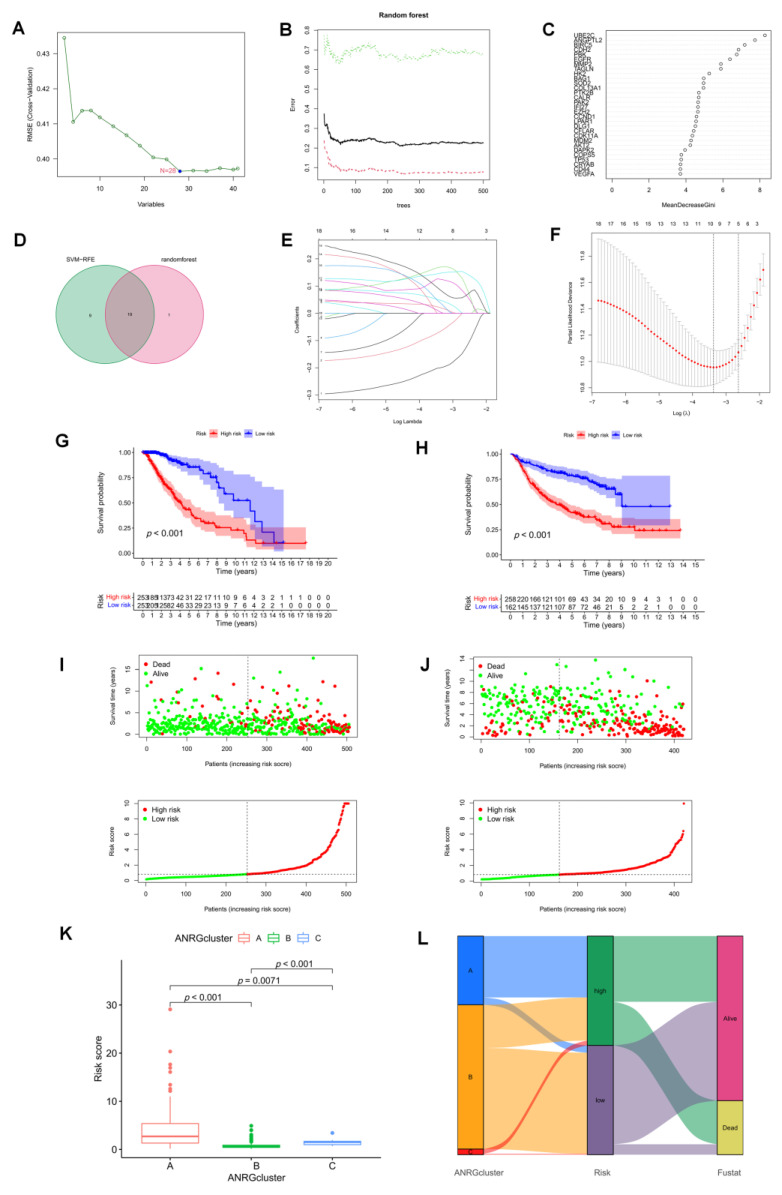
Identification of prognostic features associated with Anoikis. (**A**) A plot illustrating the process of selecting biomarkers using the support vector machine-recursive feature elimination (SVM-RFE) technique. The SVM-RFE technique was used to identify a subset of 28 characteristics from the DEGs. (**B**) The effect of the decision tree number on the error rate. The X-axis denotes the number of decision trees, while the y-axis shows the error rate. When approximately 240 decision trees are used, the error rate is generally steady. (**C**) The Gini coefficient method results in a random forest classifier. The x-axis displays the genetic variable, and the y-axis the significance index. (**D**) Venn diagram showing the feature genes shared by random forest, and SVM-RFE algorithms. (**E**) LASSO analysis with 10-fold cross-validation identified seven prognostic genes. Each curve corresponds to one gene. (**F**) Coefficient profile plots of seven prognostic ANRGs. Vertical dashed lines are plotted at the best lambda. (**G**,**H**) The KM curves showed a different prognosis in the subtype risk group. (**I**,**J**) Risk plots were used to illustrate the survival status of each sample in the TCGA and CGGA cohorts. (**K**) Risk score in 3 clusters established before. (**L**) Alluvial diagram of subtype and living status.

We used two diagnostic machine learning methods to select signature genes associated with anoikis in LGG. For the SVM-RFE algorithm, the error was minimized when the number of features was 28 (Figure 5A). For the random forest algorithm, the 20 feature genes with the largest relative importance scores were determined (Figure 4B,C). After taking the intersection set, 19 feature genes common to both the random forest and SVM-RFE algorithms were finally identified (Figure 5D). We then participated in a Lasso-penalized Cox analysis using these 19 ANRGs (*p* < 0.05, Figure 5E,F). Finally, by multivariate Cox analysis, 7 ANRGs were identified as independent prognostic factors, including ANGPTL2, BAG1, CDH2, IFI27, PTK2B, SOD2, and UBE2C. Based on their coefficients, we calculated risk scores using the following formula.

Risk score = sum of the expressions of the 7 ANRGs * respective coefficients. The correlation coefficients are shown in Supplementary Table S3. Patients in the high-risk group in the TCGA-LGG cohort had a worse prognosis, according to KM curves, which was also seen in the CGGA validation cohort (Figure 5G,H). Risk plots display specific survival results for each patient in the TCGA cohort and the CGGA cohort, showing a steady rise in mortality with increasing risk scores (Figure 5I,J). Risk scores were significantly different in the three previous subtypes (Figure 5K), with cluster A having a higher risk score (*p* < 0.01). Alluvial plots showed the association of cluster, risk, and survival status associated with ANRGs (Figure 5L).

### 3.5. Immune Infiltration in Different Risk Groups

**Figure 6:**
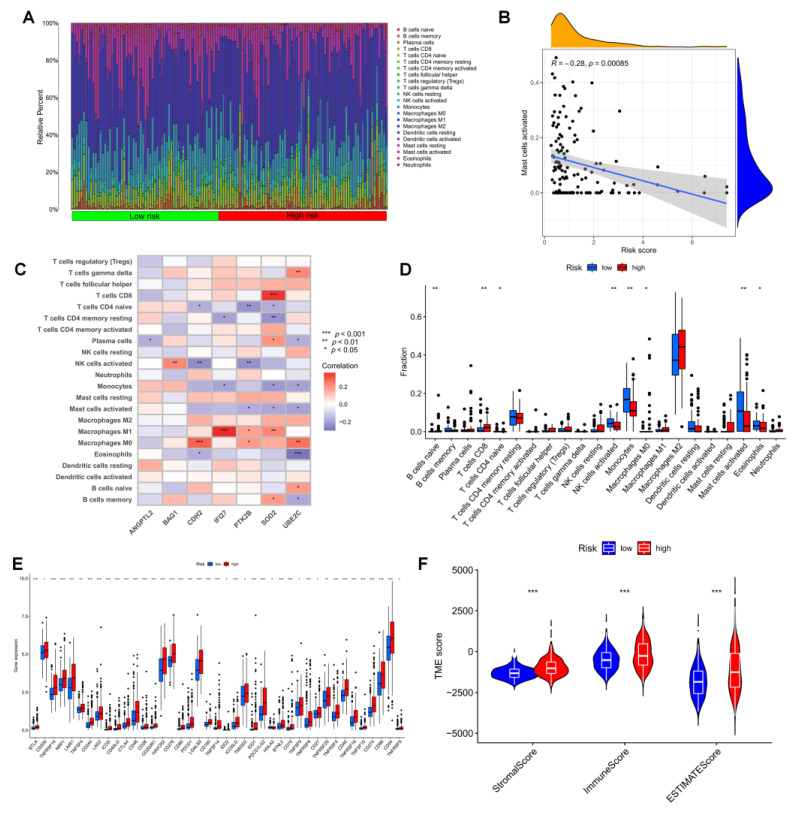
The immune microenvironment of LGG tissues with different risk scores. (**A**) Relative proportions of infiltrating immune cells for different risk subgroups. (**B**) Correlation analysis between risk scores and the proportion of activated Mast cells in LGG tissues. (**C**) Correlation between immune cells and seven hub ANRGs. (**D**) Differences in immune cell composition between the high-risk and low-risk groups. (**E**) Expression of all immune checkpoint genes in the risk group. (**F**) Estimate score of the expression profile in the high-risk group and low-risk group. * *p* < 0.05, ** *p* < 0.01, *** *p* < 0.001.

The development of gliomas and the effectiveness of immunotherapy are both significantly influenced by the immune microenvironment. To achieve this, we looked more closely at the tumor microenvironment (TME) of LGG patients. The relative proportions of invading immune cells in the high-risk and low-risk groups were measured using the “CIBERSORT”. First, the risk scores for the LGG samples were ranked from low to high to display the proportion of various immune cells (Figure 6A). With an increasing risk score, the proportion of mast cells gradually increased (R = 0.23, Figure 6B). In particular, SOD2 was highly associated with the infiltration of M1 macrophages and CD8 + T cells. The seven genes utilized to build the risk score were strongly connected with numerous immune cells (Figure 6C). The infiltration of monocytes and mast cells was greater in the low-risk group (Figure 6D). This shows that mast cell suppression may play a significant role in the poor prognosis for LGG. We discovered that practically all immune checkpoints, including CTLA-4, HAVCR2 (TIM3), PDCD1 (PD-1), TIGIT, and CD70, displayed greater activity in the high-risk group by comparing immune checkpoint activation between various risk groups (Figure 6E). Additionally, we were able to determine the stromal score and immunological score of the high-risk and low-risk groups using the ESTIMATEscore of the expression profile (Figure 6F).

### 3.6. Establishment of a Prognostic Nomogram for LGG Patients

**Figure 7:**
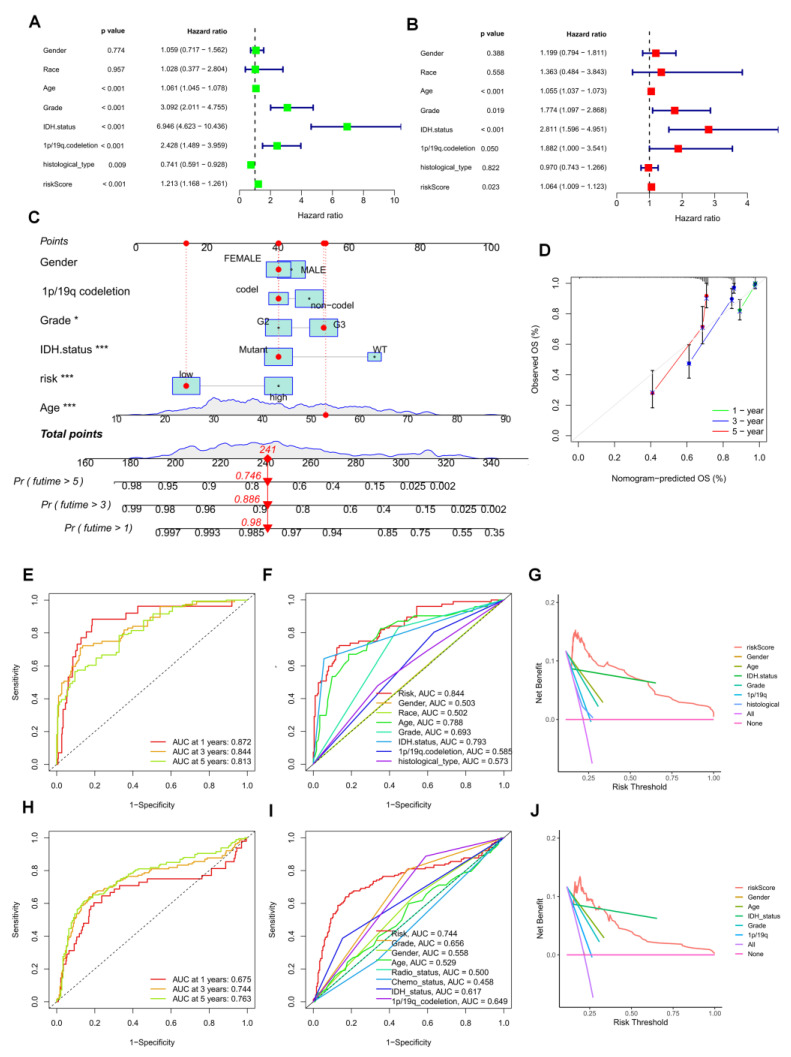
Prognostic value of risk scores in patients with LGG. TCGA cohort (**A**) Univariate and (**B**) multi-variate COX analysis to assess risk scores and clinical features (including age, grade, gender, IDH status, and 1p/19q. codeletion). (**C**) Nomogram of risk groupings and clinical characteristics predicting 1-, 3-, and 5-year survival. (**D**) Calibration curves tested for agreement between actual and predicted outcomes at 1, 3and5 years. AUC values for (**E**) TCGA and (**H**) CGGA cohort risk groupings at 1, 3, and 5 years. AUC values for (**F**) TCGA and (**I**) CGGA cohort risk groups and clinical characteristics at 3 years. DCA curves of risk scores and clinical characteristics for the (**G**) TCGA and (**J**) CGGA cohorts at 3 years. * *p* < 0.05, *** *p* < 0.001.

The risk score was identified as an independent predictive factor for LGG in the TCGA population by univariate and multivariate Cox analyses (Figure 7A,B). Then, we included information about risk groups, IDH mutation status, 1p/19q deletion status, tumor grade, age, grade, and tumor grade in the nomogram (Figure 7C). To evaluate the consistency between the prognostic model’s predicted overall survival (OS) and the actual overall survival, calibration plots were created. The findings revealed that the nomogram’s predictions were accurate (Figure 7D). The efficacy of the created model in accurately predicting OS in LGG patients was evaluated using time-dependent ROC curves. Concerning predicting OS in the TCGA cohort, the risk score did well (AUCs for 1-year, 3-year, and time-dependent ROC curves were used to assess the accuracy of the developed model for predicting OS in LGG patients. The risk score did well in the TCGA cohort at predicting OS in these people (AUCs for 1-year, 3-year, and 5-year OS: 0.872, 0.844, and 0.813; Figure 7E). Comparable outcomes were seen in the CGGA cohort (Figure 7H). In both the TCGA and CGGA cohorts, the three-year area under the curve (AUC) of the risk score was larger than the AUC of other clinicopathological characteristics (Figure 7F,I). The three-year DCA curves showed that the risk score was a good predictor of survival in LGG patients (Figure 7G,J).

**Figure 8:**
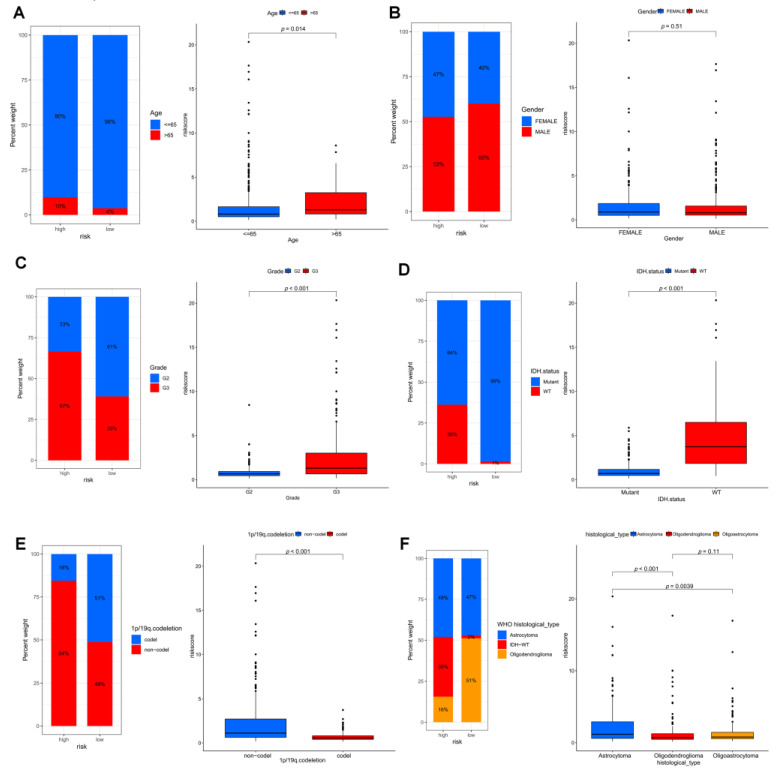
Differences in risk scores among different clinical characteristics subgroups in the TCGA cohort. (**A**) age, (**B**) gender, (**C**) grade, (**D)** IDH mutation status, (**E**) 1p/19q. codeletion and (**F**) histological type of 2021 WHO.

Based on these observations, we compared in detail whether risk scores differed across subgroups of clinical characteristics. We found that individuals with older age, G3 stage, no mutation in IDH, and no common defect in 1p/19q showed higher risk scores (Figure 8A–F, *p* all < 0.05).

### 3.7. Mutation Landscape in Different Risk Groups

**Figure 9:**
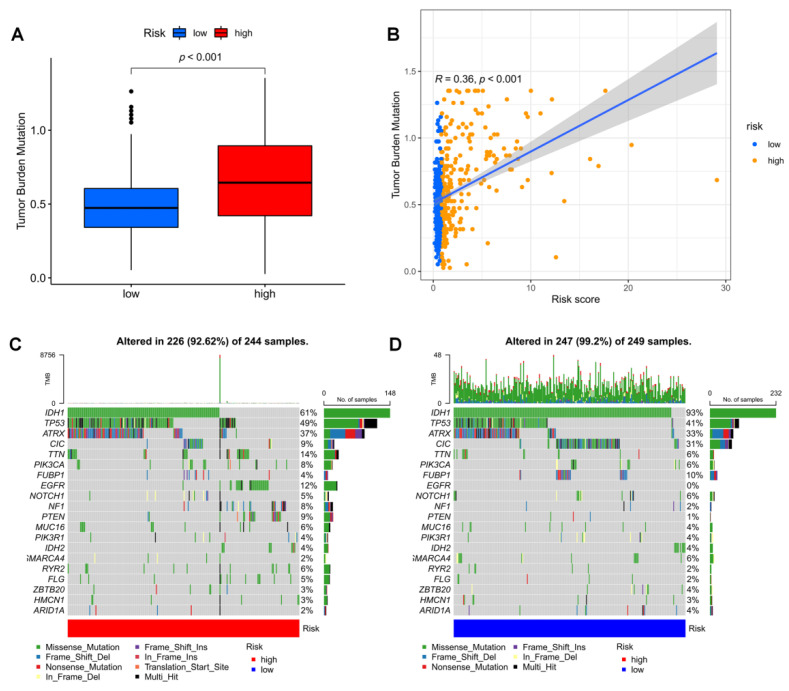
Mutation analysis based on risk score model. (**A**) Differences in TMB in high and low-risk score groups. (**B**) Correlation of risk score and TMB. (**C**,**D**) Waterfall plots summarizing the mutations in high- and low-risk patients.

Tumor mutation load (TMB) was higher in the high-risk group, according to our analysis of the relationship between risk score and TMB (Figure 9B) and the variation in TMB among various risk groups (Figure 9A). IDH1, TP53, and ATRX were the most frequently mutated genes in high-risk and low-risk groups, respectively. However, there were fewer IDH mutations and more mutations in other genes in the high-risk group. As a result, we generated two waterfall plots to explore the detailed gene mutation characteristics between high-risk and low-risk populations (Figure 9C,D).

### 3.8. Immunotherapy and ICB Response

**Figure 10:**
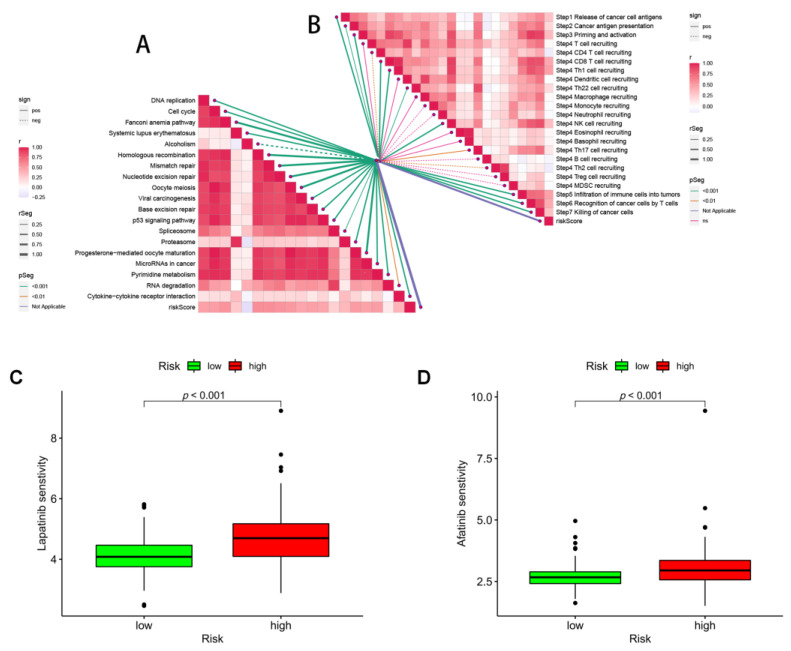
Prediction of immunotherapy and pharmacotherapy for different risk subgroups. (**A**) Correlation between risk score and ICB response signature, and (**B**) correlation between risk score and each step of the tumor immune cycle. IC50 values were calculated for patients in the high- and low-risk groups based on lapatinib (**C**) and afatinib (**D**) to assess the sensitivity of chemotherapeutic agents.

Since the immune microenvironment mediates the ICB response, we further analyzed the correlation between the risk score and the ICB response signature. We found that the risk score was significantly negatively correlated with alcoholism only, while it was significantly positively correlated with other ICB response signatures (Figure 10A). Subsequently, to further refine the immune signature of the tumor microenvironment, we also performed a correlation analysis between tumor immune cycle steps and the risk score. Once more, risk scores were significantly and favorably correlated with the majority of the critical stages of the tumor immune cycle, such as the release of cancer cell antigen (step 1), presentation of cancer antigen (step 2), priming and activation (step 3), immune cell transporting to the tumor (step 4) (CD8 T cell recruitment, Th1 cell recruitment, Th22 cell recruitment, NK cell recruitment, and Th17 cell recruitment), infiltration of immune cells into tumors (step 5), and recognition of cancer (Figure 10B).

Finally, we investigated the potential sensitivity of clinical agents in the high-risk and low-risk groups using the “pRRophetic” R package and screened some chemotherapeutic agents that could be used to treat gliomas, such as lapatinib and afatinib (Figure 10C,D). Almost all of these agents showed higher IC50 in patients with high scores (Supplementary Figure S2), indicating that patients with high-risk scores may be more sensitive.

### 3.9. Correlation Analysis of ANRGs and Tumor Immune Microenvironment

**Figure 11:**
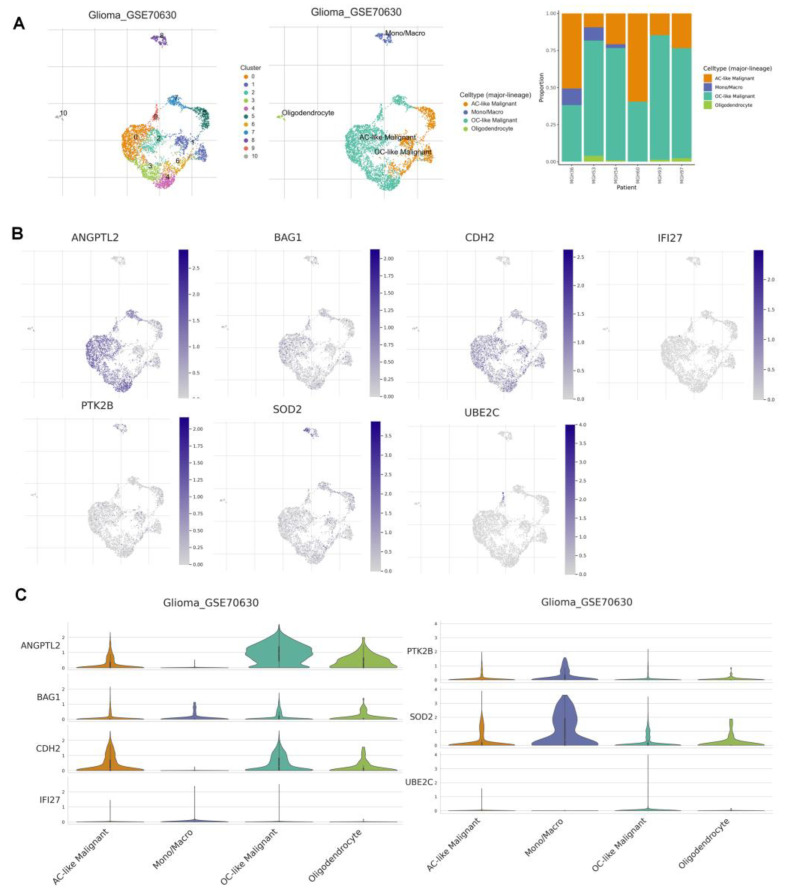
7ANRGs in single-cell RNA sequencing (**A**) Annotation of all cell types in GSE70630 and the percentage of each cell type. (**B**,**C**) Expression of ANGPTL2, BAG1, CDH2, IFI27, PTK2B, SOD2, and UBE2C in each cell type.

To examine the expression of seven ANRGs in TME, we used the single-cell data set GSE70630 of oligodendroglioma from the TISCH database. There are 10 cell populations and 4 intermediate cell types in the GSE70630 dataset, and the image depicts their distribution and number (Figure 11A). PTK2B and SOD2 were mainly expressed in monocyte macrophages. In contrast, ANGPTL2, BAG1, and CDH2 were mainly expressed in cancer cells and oligodendrocytes (Figure 11B,C).

## 4. Discussion

The prognosis for glioma patients does not significantly improve despite breakthroughs in surgery, radiation therapy, chemotherapy, and other treatments. Glioma is the most prevalent kind of malignant brain tumor in adults [28,29]. Glioma cells can penetrate along blood vessels and invade surrounding normal brain tissue, making it difficult to remove the tumor as a whole [30]. Once a low-grade glioma differentiates into a high-grade malignant glioblastoma, the invasive ability will be enhanced, and it can infiltrate and metastasize through the normal tissue space [31]. However, due to the heterogeneity of gliomas and the lack of sustained response, targeted therapies for LGG patients are less effective, and therefore there is an urgent need for more tumor metastasis-related markers for early glioma treatment to improve diagnostic accuracy.

In the absence of extracellular matrix (ECM) attachment or when cells adhere to inappropriate sites, cells undergo a specific type of apoptosis called anoikis [32]. Failure to properly execute the anoikis program may lead to rapid cell proliferation at ectopic sites. This dysregulation of apoptotic execution is becoming a hallmark of cancer cells and contributes to their metastasis to distant organs [33].

The crucial process by which epithelial cells transform into mesenchymal cells and lose their cell polarity and adhesion is known as oncogenic EMT. EMT has recently been discovered in glioma stem cells to directly impact migration, invading ability, and radiation resistance in gliomas [34]. One of the hallmarks of EMT is the resistance of tumor cells to anoikis. The development of new cancer treatment modalities to address tumor resistance to anoikis has become a hot topic of research in recent years [35,36]. Gliomas have anoikis-resistant properties that enhance their invasion of the adjacent brain parenchyma and eventually recur despite the use of standard therapies. Further exploration regarding the mechanisms of anoikis in gliomas remains to be done. A recent study found that activation of anoikis in glioma cells was associated with inhibition of p21-activated kinase 4 (PAK4) [37]. In addition, Jiang et al. found that MNX1 was bound to the upstream regulatory region of TrkB as a transcription factor to activate its expression, enhancing the ability of glioma cells to evade anoikis [30].

In this work, we found seven genes—ANGPTL2, BAG1, CDH2, IFI27, PTK2B, SOD2, and UBE2C—that together make up robust risk score characteristics. In previous studies, many correlations between these ANRGs and tumorigenesis as well as pathogenesis have been extensively reported. Increased ANGPTL2 expression in colorectal cancer (CRC) cells improves the β-catenin pathway signaling and boosts tumor cell proliferation. ANGPTL2 regulates epithelial regeneration and intestinal immune response [38]. In ovarian cancer, ANGPTL2 can even reduce peritoneal metastasis of tumor cells by inhibiting anoikis resistance [39]. BAG1 is a multifunctional protein associated with a variety of cellular processes, such as apoptosis, proliferation, growth, and motility [40]. As an autophagy-related gene, BAG1 is also considered to be an important prognostic factor in low-grade gliomas [41]. In colon cancer, knockdown of the neurotrophic factor BDNF suppresses the expression of the mesenchymal marker CDH2 leading to anoikis and immune resistance in tumor cells [42]. Atypical EGFR signaling in glioblastoma activates the transcription factor IRF3, leading to the expression of IFI27, which often plays an important oncogenic role [43]. Acute lymphoblastic leukemia (ALL) contains multiple activated kinase and cytokine receptor signatures, such as genomic alterations in PTK2B [44]. Normal cells require adherence to the extracellular matrix to survive. Cell shedding leads to a dramatic increase in reactive oxygen species (ROS), which promotes anoikis [45,46]. Mammary epithelial cells can reduce anoikis by increasing mitochondrial antioxidant enzyme SOD2 to reduce ROS produced by mitochondrial glucose oxidation [47]. Similarly, in ovarian cancer cells, SOD2 protein expression is associated with increased oxidative stress, and ovarian cancer cells rapidly increase their mitochondrial antioxidant capacity through this mechanism as a means to adapt to the loss of anchor points and escape anoikis [48]. Ma et al. found that the ubiquitin-binding enzyme E2C (UBE2C) was a key regulator of cell cycle progression and an important factor in the malignant progression of astrocytic tumors [49]. Meanwhile silencing of UBE2C in glioma leads to significant inhibition of the PI3K-Akt-mTOR pathway, while avoiding autophagy [50].

To evaluate the status of anoikis, we utilized unsupervised cluster analysis to divide LGG patients into three subgroups (clusters A, B, and C) based on 27 ANRGs. The majority of the ANRGs were discovered to be highly expressed in cluster A, which is likely what caused the individuals in group A to have a worse prognosis. The results imply that anoikis can affect how LGGs form. The two clusters of AB differed in tumor infiltration and metastasis-related pathways, according to GSVA. In the current study, both the training and validation cohorts of LGG patients showed that the anoikis-related gene signature correctly predicted OS. This gene signature was an independent predictor of LGG prognosis in both the TCGA and CGGA cohorts when considering relevant clinical characteristics, such as tumor grade, age, and sex. Clinical variables with high-risk scores tended to be statistically significant risk factors for prognosis, suggesting that the ANRG gene signature could be a predictor of prognosis and could be a proxy for prognosis. Patients with concurrent risk scores tended to have higher tumor grade, IDH-wild type, and no 1p/19q co-deletion, which was consistent with previous studies [51,52] and more suggestive of a high-risk adverse prognostic profile.

To investigate the prognostic mechanism of this feature and to provide clues for predicting immune cell infiltration (ICI), we compared the high-risk and low-risk groups in terms of the proportion of 22 immune cells, TME, gene mutations, and TMB. Consistent with previous studies, CD8+ T-cell infiltration was greater in the high-risk group. Furthermore, SOD2, among the seven risk genes, had the highest correlation coefficient with CD8 + T cells [53]. Thus, SOD2 activation of the CD8 + T cell axis may be an interesting pathway. We also found that the high-risk group exhibited higher TMB than the low-risk group, but the low-risk group expressed more high-frequency IDH and CIC mutations [54]. The high-risk group showed higher TMB, which would lead to more neoantigens and enhanced T-cell recognition, and therefore could be a good predictor of the effect of ICB therapy.

In 2013, Chen and Mellman introduced the concept of tumor immune cycling. Tumor immunity arises as a continuously self-derived cyclic process, through which immune stimulatory molecules are accumulated to amplify T cell responses [55]. Thus, the cancer-immune cycle represents the immune response of the human immune system to cancer. Immune checkpoint inhibitors, particularly treatments such as anti-PD-1/PD-L1 and CTLA-4, are effective against a wide range of tumors but have performed poorly in clinical trials in glioma [56]. The efficacy of immunotherapy in glioma is related to its unique molecular alterations, immune checkpoint expression levels and immune microenvironment. Immune cells and associated stromal components recruited and activated by tumor cells, which form tumor suppressive inflammatory TME from the early stages of tumor colonization or growth, can well hinder tumor development [57]. In addition, in patients in the high-risk group, we found that upregulation of suppressive immune checkpoint molecules, which can reduce immune cell activity, is another major feature of inflammatory TME [58]. Patients in the high-risk group tend to have higher immune checkpoint gene expression, while we found that higher risk scores correlate significantly with both tumor immune cycle and ICB response. For these high-risk subgroups of LGG patients, a combination of immunotherapeutic strategies targeting TME, remodeling of the positive immune microenvironment, and multi-targeted immunotherapeutic agents can be used to significantly improve the prognosis and generate a comprehensive response in LGG patients.

## 5. Conclusions

In conclusion, our 7 ANRGs signature can well predict the survival of LGG patients, and it will assist clinicians in creating various treatment plans. The DCA curve also indicates that LGG patients can benefit from the nomogram created using the 7 genes signature. In practical practice, columnar maps based on this concept can aid doctors in creating personalized treatments. Our study still has some inherent problems, though. Future experimental confirmation is required as all of these conclusions came from bioinformatics research.

## Data Availability

The datasets analyzed in this study can be found in the TCGA-LGG project (http://www.cancer.gov/tcga, accessed on 20 August 2022), CGGA database (http://www.cgga.org.cn/, accessed on 20 August 2022), and GTEx project (https://gtexportal.org/home/, accessed on 20 August 2022).

## Supplementary Materials

The following supporting information can be downloaded at: https://www.mdpi.com/article/10.3390/brainsci12101349/s1, Figure S1: GSVA analysis of clusters B (or A) and C; Figure S2: A drug sensitivity analysis. Table S1: 358 ANRGs from the Genecards and Har-monizome portals; Table S2: Results of univariate Cox regression analysis and survival analysis for the 57 ANRGs; Table S3: Correlation coefficients of the 7 ANRGs.
