# Supplementary figures and images for: A Bioinformatics-Based Analysis of an Anoikis-Related Gene Signature Predicts the Prognosis of Patients with Low-Grade Gliomas
Source: Brain Sci. 2022 Oct 5;12(10):1349. doi: 10.3390/brainsci12101349 (PMC9599312; doi:10.3390/brainsci12101349)

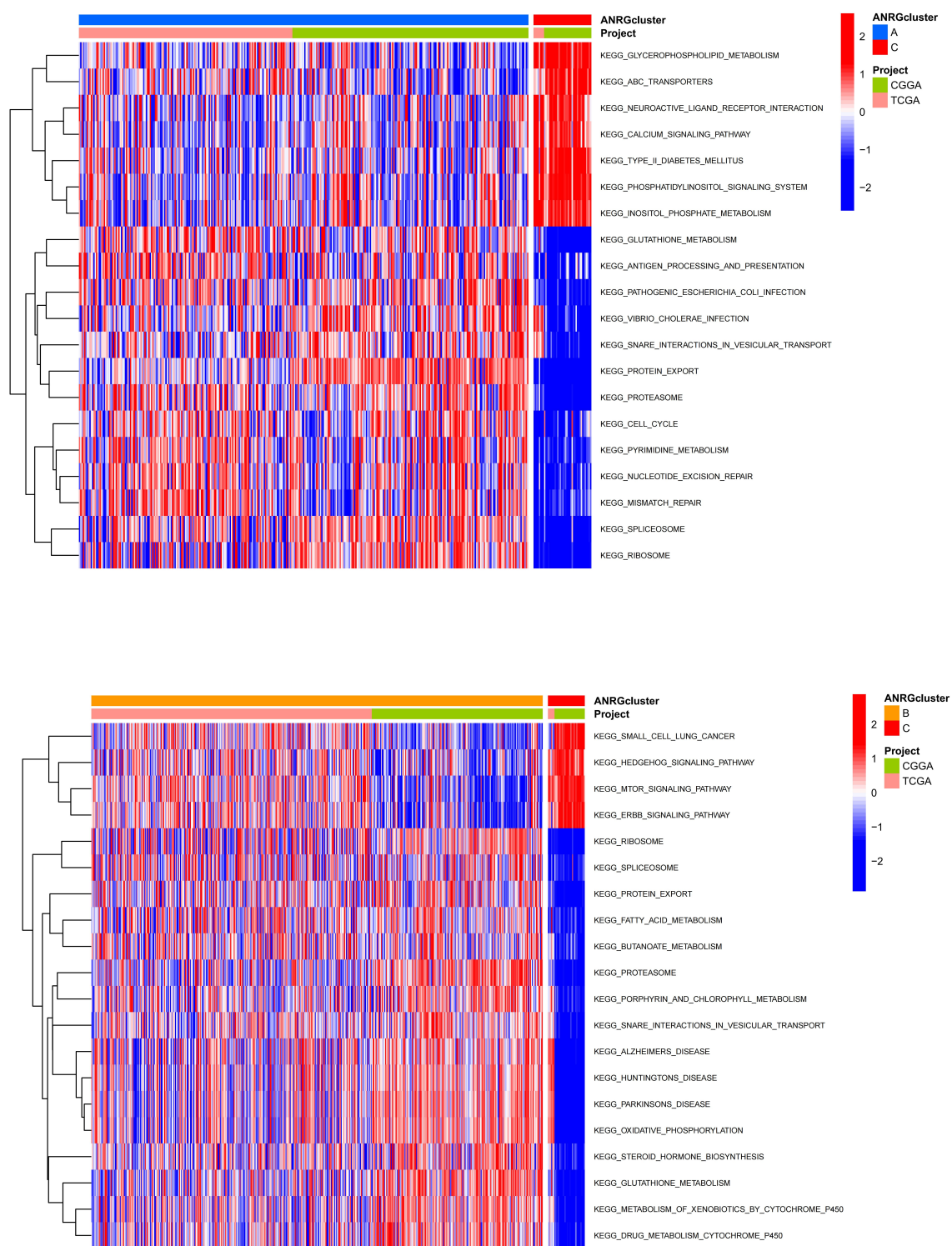

Figure S1: GSEA analysis of clusters B (or A) and C.

Supplement: Supplementary file 1 [file brainsci-12-01349-s001.zip › Figure S1.pdf]

# drugSensitivity

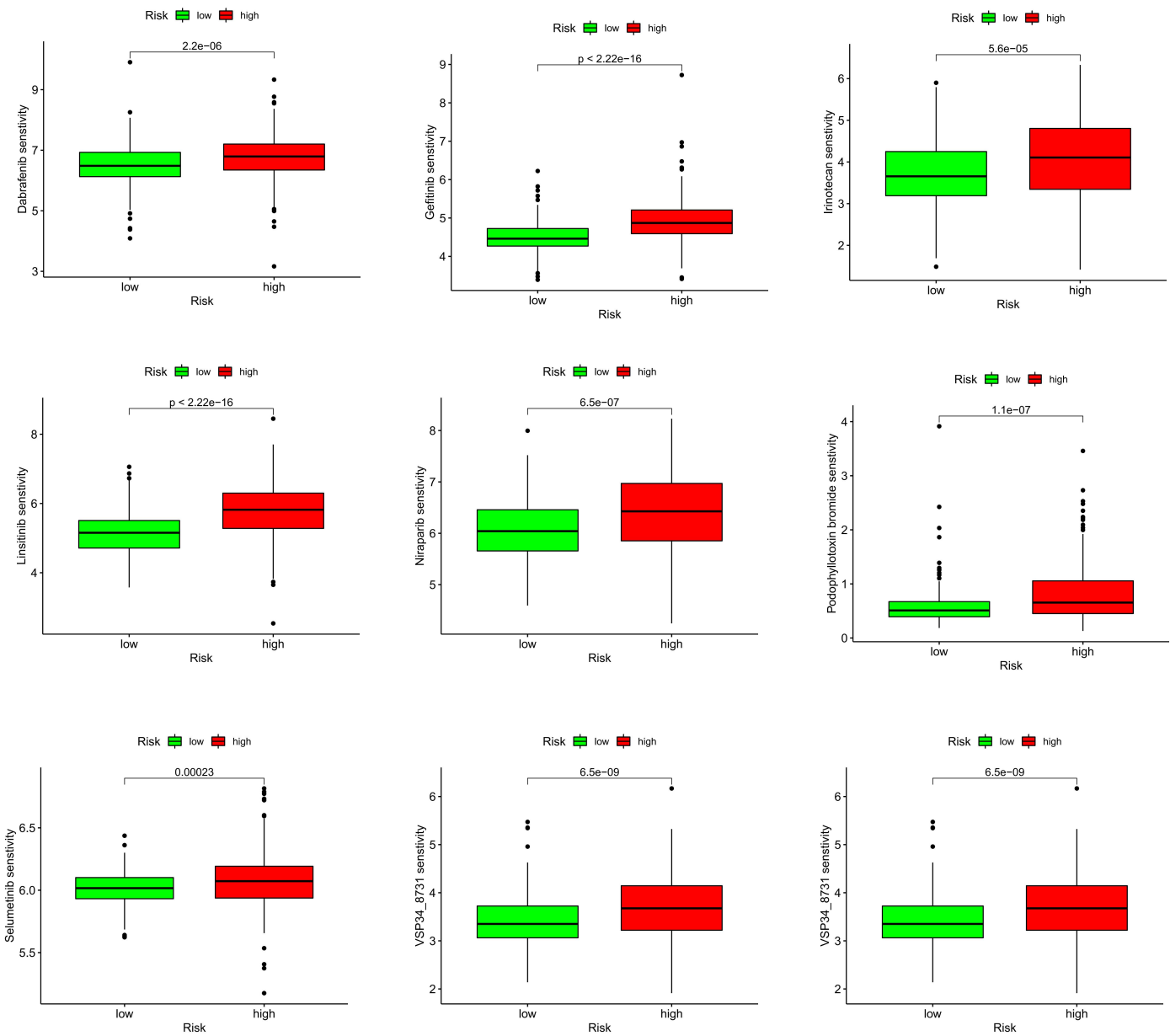

Figure S2: A drug sensitivity analysis.

Supplement: Supplementary file 1 [file brainsci-12-01349-s001.zip › Figure S2.pdf]
